# Physician emigration from Germany: insights from a survey in Saxony, Germany

**DOI:** 10.1186/s12913-018-3142-6

**Published:** 2018-05-09

**Authors:** Birte Pantenburg, Katharina Kitze, Melanie Luppa, Hans-Helmut König, Steffi G. Riedel-Heller

**Affiliations:** 1 Institute of Social Medicine, Occupational Health and Public Health, University of Leipzig, Philipp-Rosenthal-Str. 55, 04103 Leipzig, Germany; 2 State Office of Tax and Finance (Landesamt für Steuern und Finanzen), Occupational Health Management, Stauffenbergallee 2, 01099 Dresden, Germany; 3 Department of Health Economics and Health Services Research, University Medical Centre Hamburg-Eppendorf, Martinistr. 52, 20246 Hamburg, Germany

**Keywords:** Physician migration, “push” factors, job satisfaction, physician attrition

## Abstract

**Background:**

Physician migration has been gaining attention worldwide. In Germany, physician migration became a topic of interest in the context of the discussion about a shortage of physicians, for which one contributing factor may be physicians leaving the country. However, there is a lack of literature on “push” factors causing German physicians to leave. The present study seeks to provide current data in an effort to promote the identification of “push” factors motivating German physicians to emigrate.

**Methods:**

In a cross-sectional survey, all physicians ≤40 years of age registered with the State Chamber of Physicians of Saxony, Germany (*n* = 5956) were sent a paper-pencil questionnaire examining socio-demographics, job satisfaction, the wish to emigrate, and the likelihood of moving abroad in the near future. Variables associated with the wish to emigrate were assessed with multivariate logistic regression models.

**Results:**

Approximately 30% of participants wished to emigrate. The favourite destination countries were Switzerland, Scandinavian countries, and Australia or New Zealand. Of participants wishing to emigrate, approximately 52% thought it likely to emigrate for a limited, and 15% for an unlimited period of time. Participants with the wish to emigrate were significantly less satisfied with their job situation as compared to physicians without the wish to emigrate, the one exception being their “relationship with patients”. The three aspects with the highest difference in satisfaction were the overall work situation, followed by work load, and time for family, friends, and leisure activities. Being a woman, being in a relationship, and having children were associated with a lower chance for wishing to emigrate. Higher satisfaction with the factors “work load”, “patient care”, and “structural aspects” was also associated with a lower chance for wishing to emigrate.

**Conclusions:**

Emigration seems to be a viable option for at least a subset of physicians. Preventive measures should address modifiable determinants associated with an increased chance for wishing to emigrate, such as job satisfaction. Especially satisfaction with the factor “work load” seems to play a crucial role as a “push” factor for physician emigration.

**Electronic supplementary material:**

The online version of this article (10.1186/s12913-018-3142-6) contains supplementary material, which is available to authorized users.

## Background

In recent years, the issue of physician migration has been gaining increasing attention worldwide [1, 2]. A main focus tends to be on physicians migrating from low- and middle-income countries, e.g. Pakistan, India, and countries in sub-Saharan Africa, to high-income countries, especially the USA and UK [3–6]. However, several studies also explore physician migration from and to European countries [7, 8], some of which, e.g. Bulgaria and Romania, also qualify as middle-income countries.

In Germany, physician migration became a topic of interest in the context of the discussion about a shortage of physicians. Although absolute numbers of physicians have been rising in Germany [9], especially in rural areas and in Eastern Germany vacancies cannot be filled, and hospitals rely on foreign physicians to maintain health care [10]. Physicians leaving the country have been discussed as one factor contributing to the lack of physicians in Germany [11, 12].

Numbers of physicians emigrating from Germany are fluctuating. In 2007, 2439 physicians left Germany, of whom 77% (*n* = 1878) had German citizenship. In 2012, the year this study was conducted, 2241 physicians left Germany, of whom 66.8% (*n* = 1497) had German citizenship. In 2014, 2364 physicians left Germany, of whom 60.5% (*n* = 1430) had German citizenship [13–15]. Switzerland, Austria, and the USA remained the three favourite destination countries [13–15]. In 2012 (and in 2014), Saxony ranked fourth in states (Bundesländer) with the lowest density of physicians [16, 17], while the percentage of emigrating physicians was similar to the national average (0.5% versus 0.6% in 2009) [12]. Thus, physician emigration may be a particularly relevant issue for the state of Saxony.

Dussault et al. [18] name a variety of factors associated with migration and choosing a destination country: “*individual … factors* that increase the probability of being mobile, such as … portability of skills, single status, fluency in the language …; *organizational factors* (real or perceived), such as heavy workload, insufficient compensation …, a dearth of career prospects …; *health care system factors*, such as … inadequacy of human resource policies …, insufficient funding of health services …; *general environmental factors*, such as poor economic conditions …, lack of security …; *other factors*, which include active recruitment by foreign agencies …”. In the context of physician migration, Mejia et al. (1979) specify that “migration is the result of the interplay of various forces, political, social, economic, legal, historical, cultural, educational, etc., operating at both ends of the migratory axis. … “push” forces, … operating in the donor country; and “pull” forces, … operating in the recipient country.” [19]. “Push factors are those that are considered to discourage physicians from remaining in a country and result in interest in leaving .... Pull factors are those that are perceived as making another country a more attractive place to practice and live” [20].

Only few studies elucidate “push” factors causing physicians to leave Germany. In 2004, a report by Ramböll management identified hierarchical structures, authoritative leadership, low income, and poor professional supervision as main reasons for German physicians leaving their country to work abroad in patient care [21]. In a qualitative study conducted among 27 medical doctors in 2010, hierarchical structures, high workload, lack of structure in postgraduate training, increasing administrative burden, incompatibility of clinical practice and research, lack of collegiality, and work-life imbalance were mentioned as reasons for moving abroad, while, in contrast to the Ramböll study, salary was not a main motivation to emigrate [22].

The present study seeks to provide current data derived from a large sample of physicians in an effort to promote the identification of “push” factors motivating German physicians to emigrate. Only if these factors are known, can measures be developed to prevent physicians from leaving the country.

As part of a larger survey on Saxon physicians’ wishes to leave patient care in Germany [23–25], the aim of the present analysis was first, to elucidate the prevalence of physicians’ wishes to emigrate; second, to gain insight into job satisfaction of physicians wishing to emigrate; and third, to identify factors associated with the wish to emigrate.

## Methods

### Study population and study design

In this cross-sectional and comprehensive survey all physicians ≤40 years of age registered with the State Chamber of Physicians of Saxony (SLÄK) (*n* = 5956) were sent paper-pencil questionnaires by mail. For the purpose of this study, an age group was chosen for which questions of moving abroad or leaving patient care were particularly relevant [26, 27]. Registration with the SLÄK is mandatory for all licensed physicians practicing medicine in Saxony or, if not practicing medicine, having their main residence in Saxony. An enclosed letter informed about the purpose of the study, that questionnaires were completely anonymous, and that completing and sending back the questionnaire was regarded as having given informed consent to study participation. From September 2012 to February 2013, 40% of questionnaires were returned (*n* = 2357). The sample contained more women (66% versus 58%, *p* < 0.001), and was slightly younger (32.9 years versus 33.2 years, p < 0.001) than the physician population in Saxony. The Ethics committee of the University of Leipzig (Ethik-Kommission an der Medizinischen Fakultät der Universität Leipzig) approved the study, including the procedure of obtaining informed consent.

### Questionnaire and instruments

The questionnaire was developed based on a review of the scientific literature and on existing instruments (an English language version of the questionnaire can be found as an Additional file 1. Note that the English version has not been validated and translation did not follow standardized translation procedures. It is solely provided for the purpose of better illustrating the acquisition of data in this study). The questionnaire included questions on socio-demographics (based on [28, 29]); self-generated questions on wishes to go abroad for clinical work and how likely participants perceived their going abroad during the next 5 years (based on [21, 29]) (English translation: “How much do you currently wish you could go abroad for clinical work?”; possible answers: “1 = not at all”, “2=rather not”, “3 = don’t know”, “4 = rather yes”, “5 = absolutely”; and “How likely do you think it is that you will go abroad for a limited or unlimited period of time during the next 5 years?”; possible answers: “1 = impossible”, “2 = rather not”, “3 = indecisive”, “4 = rather yes”, “5 = definitely”). Questions addressing job satisfaction were mainly based on [30]: Participants were asked to rate their satisfaction with the overall job situation as well as with 20 different aspects of their job situation (Table 6) on a 5-point Likert scale ranging from 1 = very dissatisfied to 5 = very satisfied. The questionnaire was pretested by a sample of non-participating physicians for comprehensibility and usability.

### Data analysis

Of the 2357 returned questionnaires, 9 questionnaires were excluded: 3 persons were > 40 years of age, one physician already worked abroad, and 5 questionnaires were incomplete. For the present analysis, data of physicians who had German citizenship and who worked in patient care were included (*n* = 1701). As not all participants fully completed all questions, the percentages in the results section correspond to the number of actual answers given.

To evaluate job satisfaction, the mean of each item in the job satisfaction questionnaire was calculated and t-tests were employed to compare the satisfaction of physicians with and without the wish to emigrate. In addition, the difference between means was calculated to identify the professional aspects where physicians with and without the wish to emigrate differ most in satisfaction.

As chi^2^-tests are used to detect a “relationship between two categorical variables” when the sample size is large enough, i.e. “the expected value of each cell is five or higher” [31] it was employed to compare the perceived likelihood to emigrate in the near future between participants with and without the wish to emigrate.

Multivariable logistic regression analyses are used for “statistical models in which there are multiple independent” variables and a dichotomous outcome variable [32]. It was therefore performed to identify variables associated with the wish to emigrate. Job satisfaction was included in the regression analysis in the form of 5 main factors derived from factor analysis (principal component analysis with promax rotation, Kaiser criterion of eigenvalues > 1, including items with factor loadings ≥0.5, Table 1) of the 20 different aspects of job satisfaction in the job satisfaction questionnaire: factor 1: “work environment” (work atmosphere, relationship with superiors, relationship and professional exchange with colleagues, work enjoyment, intellectual stimulation at work); factor 2: “work load” (work load, time for family, friends and leisure activities, stress level at work); factor 3: “patient care” (possibility to refer patients to specialists whenever you deem it necessary, quality of the medical care you provide, possibility to treat patients as you deem optimal); factor 4: “structural aspects” (job security, current income, equality of women and men, career opportunities) and factor 5: “humaneness” (relationship with patients, relationship with non-medical staff).

**Table 1:** Factor analysis of aspects of job satisfaction

	Factor loading				
	Factor 1: Work environment	Factor 2: Work load	Factor 3: Patient care	Factor 4: Structural aspects	Factor 5: Humaneness
Work atmosphere	0.8327	0.1659	−0.145	− 0.0449	0.1619
Relationship with superiors	0.8095	0.1111	−0.1883	0.0032	−0.0443
Relationship/professional exchange with colleagues	0.7912	−0.229	0.0473	−0.0904	0.0918
Work enjoyment	0.5361	0.2244	0.2146	−0.0615	0.1687
Intellectual stimulation at work	0.5045	−0.1679	0.39	−0.0306	−0.1581
Training opportunities	0.3216	0.1007	0.239	0.1879	−0.3813
Work load	0.0421	0.8692	0.001	−0.0259	0.0107
Time for family, friends, leisure activities	−0.072	0.8587	−0.0022	0.0191	−0.0787
Stress level at work	0.1018	0.7716	0.0425	−0.0324	0.0559
Time for administrative tasks	−0.0381	0.4966	0.2229	0.0668	−0.097
Possibility to refer patients to specialists whenever you deem it necessary	−0.1476	−0.0634	0.7658	0.0147	0.072
Quality of the medical care you provide	−0.0289	0.0393	0.7515	−0.0726	0.1389
Possibility to treat patients as you deem optimal	−0.0389	0.1589	0.7358	−0.0685	0.1789
Job security	−0.1371	−0.0401	− 0.0724	0.832	0.0398
Income	−0.1187	−0.0524	0.0259	0.6887	0.2102
Equality of women and men	0.0656	0.17	−0.1795	0.6445	0.0121
Career opportunities	0.2458	−0.0727	0.1595	0.5134	−0.3163
Social status	0.2767	−0.0623	0.1081	0.3983	0.2883
Relationship with patients	−0.0186	−0.0543	0.4173	0.0705	0.6719
Relationship with non-medical staff	0.3502	0.0558	−0.0239	0.068	0.5827

Factor analysis of aspects of job satisfaction was performed. Items with factor loadings ≥0.5 were included

Participants’ age (in years), clinical work experience (in years), and satisfaction with the factors “work environment”, “work load”, “patient care”, “structural aspects”, and “humaneness” were introduced as continuous variables. Female sex (reference category [rc]: male), being in a relationship (rc: no relationship), having children (rc: no children), former stay abroad > 3 months (rc: no former stay abroad), being a specialist (rc: no specialization), would become a physician again (rc: would not become a physician again or indecisive), working full-time (rc: part-time), working in an urban setting (rc: rural setting), working in an inpatient setting (rc: outpatient setting), having a leading position/having their own practice (rc: non-leading position/employee in a practice) were dummy coded before introducing them into the regression model. After exclusion of missing values, the analysis was performed with data of *n* = 1575 participants and reached a pseudo-R^2^ of 0.1488. All data analysis was performed with Stata 13.0 for Windows.

## Results

### Characteristics of the study population

The characteristics of the study population are detailed in Table 2.

**Table 2:** Characteristics of the study population (*n* = 1701)

	% (n)
Female sex	61.3 (1043)
In a relationship	85.2 (1446)
Having children	54.5 (927)
Former stay abroad^a^	52.9 (899)
Specialist qualification	38.0 (645)
Works full-time	83.0 (1411)
Inpatient setting	82.5 (1377)
Urban setting	77.4 (1261)
Leading position or own practice	15.2 (253)
Would become a physician again	72.1 (1225)
	M (SD)
Age (years)	32.92 (3.95)
Clinical work experience (years)	5.51 (3.75)

^a^Question: “Have you ever spent more than 3 months outside of Germany?” Percentages were calculated with the number of answers actually given and may differ from calculations based on a denominator without missing values

*M* mean, *SD* standard deviation

### Participants’ wishes to emigrate

Of all participants, 29.5% wished to emigrate (absolutely/rather yes), 55.7% didn’t wish to emigrate (not at all/rather not), and 14.9% didn’t know (Table 3).

**Table 3:** Wish to emigrate among the study population

	% (n)
Yes	29.5 (496)
Don’t know	14.9 (250)
No	55.7 (937)
Total	1683

The wish to emigrate was assessed by asking “How much do you currently wish you could go abroad for clinical work?”. Percentages don’t add up to 100% due to rounding

As expected, a higher percentage of participants who wished to emigrate as compared to those who did not wish to emigrate or didn’t know had thoroughly searched possibilities for clinical work abroad during the previous 3 years (72.9% versus 21.5%, *p* < 0.001) (Table 4).

**Table 4:** “Have you ever thoroughly searched possibilities for clinical work abroad?”

	Wish to emigrate	No wish to emigrate/ don’t know	*p*
	% (n)	% (n)	
Yes, during the last three years	72.9 (361)	21.5 (255)	**< 0.001**
Yes, more than three years ago	16.8 (83)	26.8 (318)	
No	10.3 (51)	51.7 (614)	
Total	495	1187	

Using chi^2^-test, participants with and without the wish to emigrate were compared regarding whether they had ever searched possibilities for clinical work abroad

The corresponding *p* value is shown in bold

Participants wishing to emigrate (*n* = 495) were asked how likely they perceived their going abroad for clinical work during the following 5 years either for a limited or an unlimited period of time. More than half thought it likely (definitely/rather yes) to emigrate for a limited period of time while 14.5% thought it likely (definitely/rather yes) to emigrate for an unlimited period of time (Table 5).

**Table 5:** Likelihood to emigrate for a limited or an unlimited period of time during following 5 years

	Limited period of time	Unlimited period of time
	% (n)	% (n)
Likely	51.9 (251)	14.5 (66)
Don’t know	27.9 (135)	29.6 (135)
Unlikely	20.3 (98)	55.9 (255)
Total	484	456

Participants wishing to emigrate were asked how likely they perceived their going abroad for clinical work during the following 5 years either for alimited or an unlimited period of time. Percentages don’t add up to 100% due to rounding. Percentages were calculated with the number of answers actually given in responseto the question

Participants wishing to emigrate were asked how likely they perceived their going abroad for clinical work during the following 5 years either for a limited or an unlimited period of time. Percentages don’t add up to 100% due to rounding. Percentages were calculated with the number of answers actually given in response to the question.

### Job satisfaction of participants with and without the wish to emigrate

Participants who wished to emigrate were significantly less satisfied with their job situation as a whole as compared to participants who did not wish to emigrate (Table 6). They were also less satisfied with all aspects of their job situation except for their “relationship with patients” (Table 6). The three aspects with the highest difference in satisfaction were the overall work situation (difference of − 0.51 points on 5-point Likert scale), work load (− 0.48 points), and time for family, friends, leisure activities (− 0.45 points) (Table 6).

**Table 6:** Job satisfaction of participants with and without the wish to emigrate

	Wish to emigrate (*n* = 496)	No wish to emigrate/ don’t know (*n* = 1187)	Diff.^a^	*p*
	M (SD)	M (SD)		
Overall job situation	3.05 (0.95; 2.97–3.14)	3.56 (0.90; 3.51–3.61)	−0.51	**< 0.001**
Work load	2.53 (1.07; 2.44–2.63)	3.01 (1.07; 2.95–3.07)	−0.48	**< 0.001**
Time for family, friends, leisure activities	2.16 (1.06; 2.06–2.25)	2.61 (1.11; 2.54–2.67)	−0.45	**< 0.001**
Relationship with superiors	3.50 (1.07; 3.41–3.60)	3.85 (0.96; 3.80–3.91)	−0.35	**< 0.001**
Work atmosphere	3.51 (0.99; 3.43–3.60)	3.86 (0.90; 3.81–3.91)	−0.35	**< 0.001**
Training opportunities	3.01 (1.12; 2.91–3.11)	3.35 (1.01; 3.30–3.41)	−0.34	**< 0.001**
Work enjoyment	3.44 (1.00; 3.35–3.53)	3.78 (0.85; 3.73–3.83)	−0.34	**< 0.001**
Income	3.47 (1.05; 3.37–3.56)	3.80 (0.90; 3.75–3.85)	−0.33	**< 0.001**
Possibility to treat patients as you deem optimal	2.96 (0.95; 2.88–3.05)	3.28 (0.89; 3.23–3.33)	−0.32	**< 0.001**
Career opportunities	3.05 (0.97; 2.97–3.14)	3.37 (0.94; 3.31–3.42)	−0.32	**< 0.001**
Stress level at work	2.49 (1.01; 2.40–2.58)	2.80 (1.00; 2.75–2.86)	−0.31	**< 0.001**
Equality of women and men	3.50 (1.10; 3.40–3.59)	3.80 (0.99; 3.75–3.86)	−0.3	**< 0.001**
Time for administrative tasks	2.06 (1.02; 1.97–2.15)	2.33 (1.00; 2.28–2.39)	−0.27	**< 0.001**
Social status	3.58 (0.98; 3.50–3.67)	3.85 (0.86; 3.81–3.90)	−0.27	**< 0.001**
Intellectual stimulation at work	3.36 (1.00; 3.27–3.45)	3.62 (0.90; 3.57–3.67)	−0.26	**< 0.001**
Job security	3.86 (1.04; 3.77–3.95)	4.12 (0.89; 4.07–4.17)	−0.26	**< 0.001**
Relationship with non-medical staff	3.85 (0.79; 3.78–3.92)	4.00 (0.76; 3.96–4.04)	−0.15	**< 0.001**
Possibility to refer patients to specialists whenever you deem it necessary	3.49 (0.97; 3.40–3.57)	3.63 (0.89; 3.58–3.68)	−0.14	**0.004**
Relationship/professional exchange with colleagues	3.73 (0.87; 3.65–3.80)	3.86 (0.87; 3.81–3.91)	−0.13	**0.004**
Quality of the medical care you provide	3.61 (0.71; 3.54–3.67)	3.72 (0.70; 3.68–3.76)	−0.11	**0.002**
Relationship with patients	3.89 (0.65; 3.83–3.95)	3.95 (0.65; 3.92–3.99)	−0.006	0.0562

Means of 5-point Likert scales (1 = very dissatisfied to 5 = very satisfied) were calculated and compared between participants with and without the wish to emigrate using t-test. Corresponding *p* values are shown

^a^ Difference in mean satisfaction between participants with and without the wish to emigrate

Corresponding *p* values are shown and significant *p* values printed in bold

### Variables associated with the wish to emigrate

Female sex, being in a relationship, and having children were associated with a lower chance for wishing to emigrate (Table 7). Higher satisfaction with the factors “work load”, “patient care”, and “structural aspects” was also associated with a lower chance for wishing to emigrate (Table 7). In contrast, a former stay abroad was associated with a higher chance for wishing to emigrate.

**Table 7:** Variables associated with the wish to emigrate

	Wish to emigrate		
Independent variable	OR [SE]	CI 95%	*p*
Female sex	0.62 [0.08]	0.47–0.80	**< 0.001**
Age	1.10 [0.03]	1.03–1.17	**0.004**
In a relationship	0.67 [0.11]	0.48–0.94	0.019
Having children	0.48 [0.07]	0.36–0.64	**< 0.001**
Former stay abroad	2.76 [0.36]	2.15–3.55	**< 0.001**
Clinical work experience	0.92 [0.04]	0.85–0.99	**0.022**
Specialist qualification	0.91 [0.19]	0.60–1.37	0.655
Would become a physician again	0.94 [0.13]	0.71–1.24	0.667
Works full-time	1.50 [0.31]	1.01–2.24	**0.044**
Urban setting	1.37 [0.22]	1.00–1.87	0.050
Inpatient setting	1.26 [0.26]	0.85–1.89	0.253
Leading position or own practice	1.00 [0.23]	0.63–1.56	0.986
Satisfaction with			
- work environment	0.82 [0.09]	0.66–1.02	0.075
- work load	0.70 [0.06]	0.60–0.82	**< 0.001**
- patient care	0.77 [0.08]	0.63–0.95	**0.016**
- structural aspects	0.60 [0.06]	0.49–0.74	**< 0.001**
- humaneness	1.13 [0.13]	0.90–1.41	0.307

Multivariate logistic regression analysis was performed to determine variables associated with the wish to emigrate

Corresponding *p* values are shown and significant *p* values printed in bold

*OR* odds ratio, *SE* standard error, *CI* confidence interval

### Destination countries favoured by participants wishing to emigrate

When asked “Which country, apart from Germany, would you most likely choose for working in patient care?” most participants wishing to emigrate favoured Switzerland, followed by Scandinavian countries, and Australia or New Zealand (Table 8).

**Table 8:** Destination countries favoured by participants with the wish to emigrate

Destination country	% (n)
Switzerland	23.2 (115)
Sweden/Norway/Denmark/Scandinavian country	15.9 (79)
Australia/New Zealand	9.7 (48)
Great Britain/Ireland	7.1 (35)
USA/Canada	6.9 (34)
France	4.2 (21)
Botswana, Burkina Faso, Capo Verde, Tanzania, West Africa, South Africa, Africa	3.8 (19)
Austria	3.2 (16)
Argentina, Brazil, Caribbean, Chile, Columbia, Mexico, Middle- or South America	2.8 (14)
Developing country/Developmental aid/Doctors without Borders	1.2 (6)
Belgium/Netherlands/Luxemburg	1.2 (6)
Spain	1.2 (6)
Thailand/India/Nepal/China	1.0 (5)
Italy	0.6 (3)
Dubai/Qatar	0.4 (2)
Romania	0.2 (1)
Not specified	17.3 (86)
Total	496

## Discussion

In the present sample approximately 30% of participants wished to work abroad in patient care. Nationwide, 0.5% of all working physicians with German citizenship emigrated in 2012 (calculations based on [14, 16, 33]) and 0.6% in 2013 (calculations based on [34–36]). Two previous studies looked at rates of physicians contemplating emigration: among specialists in Iceland, 67% had considered emigration [37]. Of clinically active Canadian family physicians, “3.0% planned to relocate to other countries within” 2 years after the study was conducted in 2004, while the actual migration rate among family physicians during these years was 0.5% [38] and thus, similar to numbers observed in Germany. Differences regarding population characteristics, especially age, need to be taken into account when comparing the results: while the present study included participants up to 40 years of age and thus, still relatively flexible individuals, nationwide numbers for Germany comprise physicians of all age groups, of which especially the older, more established physicians are less likely to consider moving abroad. Similarly, the average age of the Canadian study sample was 47.4 years (15 years older than the sample from Saxony), and only 5% of physicians in the Icelandic sample were 39 years or younger.

Although the results of the present study, together with available numbers for Germany, do not indicate a broad outflow of physicians, they do suggest that emigration is a viable option for at least a subset of physicians. This is supported by the finding that the vast majority of those wishing to emigrate had actually taken recent action to gather information about job opportunities abroad.

While more than half of those wishing to emigrate found a temporary stay abroad likely, only 15% found it likely to emigrate permanently. Relief about these results is out of place: favourable professional and personal factors encountered abroad may encourage migrants to stay [39]. Among UK-trained physicians in New Zealand, only 30% of participants had planned to emigrate indefinitely, however, at the time of the study 89% intended to stay [40]. In addition, even those willing to return might be prevented from doing so when encountering barriers such as unchanged unfavourable work conditions [41], complicated recognition of qualifications acquired abroad [40], or no longer fitting into the culture of origin [42].

Nearly a third of German participants were unsure about their wish to emigrate. Attention must be paid to them: the undecided today may become emigrants tomorrow depending on the professional and private conditions they encounter in the near future.

Physicians wishing to emigrate exhibited lower job satisfaction, except for their “relationship with patients”. This is in contrast to previous observations made in the context of this study on physicians’ wishes to leave: those who wished to leave patient care were also less satisfied with their relationship with patients [23]. Therefore, physicians wishing to work abroad in patient care do not seem to be frustrated with clinical work itself, but rather with the structure in which to provide patient care.

Physicians with a wish to emigrate showed the biggest difference in satisfaction in professional aspects such as overall job situation, work load, and time for private life. This was followed by relationship with superiors, work atmosphere, training opportunities, work enjoyment, and income. Thus, the present results partially confirm “push” factors identified in previous studies [21, 22].

Being a woman, being in a relationship, and having children were associated with a decreased chance for wishing to emigrate. While no association between sex and considering emigration was found among Icelandic specialists [37], among Canadian physicians, male sex and single status were associated with an increased chance for planning migration to another country [38]. These findings make sense as the logistics of moving a family, which may require finding a job for the spouse, day care or school for the children, are more complicated than moving alone. Having children may also increase the wish to live close to other family members such as grandparents. Why men are more likely to wish to emigrate remains speculative: perhaps for them working abroad as a career boost is more important, maybe women are more involved in social obligations close to home and therefore less geographically flexible. Other reasons connected with gender roles are conceivable. In the present study higher age was positively associated with the wish to emigrate, while among Canadian and Icelandic physicians younger age was positively associated with considering migration [37, 38]. As the Canadian and Icelandic samples were older than the present sample, it is possible that in all three samples approximately the same age, speculatively the mid to late thirties, presents the strongest association with the wish to emigrate.

A former stay abroad was an important determinant for an increased chance for wishing to emigrate. Similarly, Icelandic physicians working abroad during vacations were more likely to consider emigration [37]. This may simply reflect a personality type, which is characterized by an increased willingness to go abroad. However, a previous stay abroad can also lower barriers connected to emigration: language skills are a prerequisite for being able to work in a specific country, and an established professional or private network may facilitate or enable organization of a work stay. As travelling, studying, and interning in foreign countries become more and more common, it is conceivable that the percentage of physicians wishing to work abroad will also increase in the near future. Thus, there is an urgent need for developing appropriate measures that prevent time-limited work experiences abroad to turn into indefinite emigration.

In the present study, higher satisfaction with the factors “work load”, “patient care” and “structural aspects” was negatively associated with wishing to emigrate. This confirms findings among Canadian and Icelandic physicians in which higher job dissatisfaction was associated with plans or consideration of moving abroad [37, 38]. Attention should be paid to the factor “work load”, comprising the aspects work load, time for family, friends, and leisure activities, and stress level at work, as a potentially important “push” factor: Higher satisfaction with “work load” was also associated with a lower chance for wishing to leave patient care [23], and with fewer symptoms of burnout in the same sample of physicians [25]. In turn, symptoms of burnout were associated with a higher chance for wishing to emigrate [25]. Thus, establishing structures for positive management of “work load” may be key for preventing physician attrition in Germany.

When asked which country they would most likely choose for clinical work, Switzerland was the favourite destination country of German physicians, followed by Scandinavian countries, Australia/New Zealand, and Great Britain.

This is somewhat different from the countries physicians actually migrated to in 2012: Switzerland, Austria, and the USA [14]. Differences between destination countries participants wish to emigrate to and those to which physicians actually emigrate may be due to practicality issues when organizing the actual migration process. E.g., for German physicians language barriers may be much lower when moving to Switzerland or Austria as compared to Scandinavia or New Zealand.

Previous studies and reports provide some insight regarding the attractiveness of specific countries in comparison to Germany, and hint to “pull” factors. Again, the factor “work load” seems to be central for the attractiveness of many destination countries: apart from the relatively low language barrier, Switzerland offers higher salaries and lower taxes [43]. In Sweden, German physicians worked around 8 h less per week and showed lower work stress [44]. Similarly, physicians in Norway showed higher life and job satisfaction, part of which is probably due to more acceptable work hours [45].

The same applies to Australia, where physicians worked around 4 h less per week, and a higher percentage was satisfied with their job [46]. England provides better structured and systematic postgraduate training, flatter hierarchies, and a higher value on teaching [47]. US physicians had higher job satisfaction, which the authors partially attribute to higher “flexibility of terms and more freely negotiable contracts as well as a more collaborative working culture” as compared to non-negotiable salaries and “rigid hierarchical working cultures” in Germany [48]. Austria, curiously both an important source and destination country for physician migration to and from Germany, offers better reconciliation of work and family, better quality of life, career chances, and lower work load [49].

### Limitations

The cross-sectional design does not allow for conclusions regarding causal relationships. Due to study design and anonymization, no information is available and follow-up not possible on whether participants wishing to work abroad eventually put this wish into practice. Anonymization prevented characterization of those who refused to participate in the study, which may have led to overestimating the prevalence of the wish to migrate. Also, due to participants’ age limit of ≤40 years the study does not provide any information on the wish to emigrate among older physicians. It is possible that among them the wish to emigrate is less prevalent. As “temporary” was not further specified, it is unknown whether respondents considering a temporary stay abroad think of a time frame of months or years. Despite the large sample size and the higher than average response rate, the results cannot be fully transferred to the entire physician population in Saxony: the sample was slightly younger and the proportion of women higher than in the population of Saxon doctors.

## Conclusions

The present study suggests that emigration is a viable option for at least a subset of physicians. Preventive measures should target modifiable determinants associated with an increased chance for wishing to emigrate, such as job satisfaction. Especially satisfaction with the factor “work load” seems to play a crucial role as a “push” factor for physician emigration. Importantly, an association between satisfaction with the factor “work load” and physicians’ wishes to leave patient care [23], as well as with symptoms of burnout [25], has been previously shown. Therefore, prerequisites allowing positive management of the factor “work load” seem to be urgently required. Such measures may also prevent the currently undecided from becoming emigrants in the future. In addition, in depth studies are needed to identify the advantages of specific destination countries from the perspective of German physicians. If feasible, attempts could be made to incorporate at least some of these positive properties, such as less work hours, higher emphasis on teaching, and flatter hierarchies, into the German health care system.

## Additional file

Additional file 1: “English version of the questionnaire” Please see “Methods”-section, paragraph on “Questionnaire and instruments”: … an English language version of the questionnaire can be found as an additional file. Note that the English version has not been validated and translation did not follow standardized translation procedures. It is solely provided for the purpose of better illustrating the acquisition of data in this study. (DOC 88 kb)

## Abbreviations

CI: Confidence interval
Diff: Difference
E.g: For example
M: Mean
OR: Odds ratio
Rc: Reference category
SD: Standard deviation
SE: Standard error
SLÄK: State Chamber of physicians of saxony (Sächsische Landesärztekammer)
UK: United Kingdom
USA: United States of America
