# Supplementary material for: Physician emigration from Germany: insights from a survey in Saxony, Germany
Source: BMC Health Serv Res. 2018 May 9;18:341. doi: 10.1186/s12913-018-3142-6 (PMC5944134; doi:10.1186/s12913-018-3142-6)
Supplement: Supplementary file 1 — “English version of the questionnaire” Please see “Methods”-section, paragraph on “Questionnaire and instruments”: … an English language version of the questionnaire can be found as an additional file. Note that the English version has not been validated and translation did not follow standardized translation procedures. It is solely provided for the purpose of better illustrating the acquisition of data in this study. (DOC 88 kb) [file 12913_2018_3142_MOESM1_ESM.doc]

**Questionnaire on**

**Wishes to Leave Among**

**Physicians in Saxony, Germany**

Dear Colleague,

thank you very much for choosing to complete this questionnaire! Not all questions will apply to you. We therefore ask you to exactly follow the instructions when filling in the questionnaire. Let us begin with some information about yourself.

| 1. | You are | **􀁻**_1_ | Female |
| --- | --- | --- | --- |
|  |  | **􀁻**_2_ | Male |

| 2. | How old are you? | _______years |
| --- | --- | --- |

| 3. | You are | **􀁻**_1_ | Single |
| --- | --- | --- | --- |
|  |  | **􀁻**_2_ | In a relationship |

| 4. | Do you have children who live in your household? | **􀁻**_0_ | No | **🢡** | **Continue with question 6** |
| --- | --- | --- | --- | --- | --- |
|  |  | **􀁻**_1_ | Yes | **🢡** | **Continue with question 5** |

| 5. | What is the age of the child / children? | _____ , _____, _____, _____ year(s) |
| --- | --- | --- |

| 6. | Do you have German citizenship? | **􀁻**_1_ | Yes |
| --- | --- | --- | --- |
|  |  | **􀁻**_0_ | No, my citizenship is: |
|  |  | ______________________________ | |

| 7. | Have you ever spent more than 3 months outside of Germany?  (Multiple answers possible) | | |
| --- | --- | --- | --- |
| **􀁻**_0_ | No | **􀁻**_2_ | Yes, for clinical work |
| **􀁻**_1_ | Yes, for medical studies | **􀁻**_3_ | Yes, for research |
| **􀁻**_99_ | Other reason: ______________________________________________________________________ | | |

Please answer the following questions regarding your job situation.

| 8. | Do you have a licence to practise medicine or a work permit? | **􀁻**_1_ | Licence to practise medicine |
| --- | --- | --- | --- |
|  |  | **􀁻**_2_ | Work permit |

| 9. | Since when? | __________ (year) |
| --- | --- | --- |

| 10. | Are you currently enrolled in / have you completed a postgraduate studies programme (e.g. Master of Public Health, Master of Business Administration)? | **􀁻**_0_ | No |
| --- | --- | --- | --- |
|  |  | **􀁻**_1_ | Yes: |
|  |  | ___________________________________ | |

| 11. | How many years of clinical work experience do you have? | **􀁻**_0_ | None |
| --- | --- | --- | --- |
|  |  | **􀁻**_1_ | ___ year(s) of clinical work experience |
|  |  |  | |

| 12. | Are you a specialist? | **􀁻**_0_ | No |
| --- | --- | --- | --- |
|  |  | **􀁻**_1_ | Yes |

| 13. | | If you are a specialist, please state your qualification(s). If you are not a specialist, please state the qualification(s) you want to acquire (multiple answers possible). | | |
| --- | --- | --- | --- | --- |
| **􀁻**_0_ | | None | **􀁻**_13_ | Microbiology, virology and infection epidemiology |
| **􀁻**_1_ | | General medicine | **􀁻**_14_ | Neurosurgery |
| **􀁻**_2_ | | Anesthesiology | **􀁻**_15_ | Neurology |
| **􀁻**_3_ | | Occupational medicine | **􀁻**_16_ | Nuclear medicine |
| **􀁻**_4_ | | Ophthalmology | **􀁻**_17_ | Orthopedics |
| **􀁻**_5_ | | Surgery | **􀁻**_18_ | Paediatrics |
| **􀁻**_6_ | | Dermatology | **􀁻**_19_ | Pathology |
| **􀁻**_7_ | | Obstetrics and gynaecology | **􀁻**_20_ | Psychiatry and psychotherapy |
| **􀁻**_8_ | | Ear, nose and throat | **􀁻**_21_ | Psychosomatic medicine and psychotherapy |
| **􀁻**_9_ | | Internal medicine | **􀁻**_22_ | Radiology |
| **􀁻**_10_ | | Dermatology | **􀁻**_23_ | Radiotherapy |
| **􀁻**_11_ | | Child and adolescent psychiatry | **􀁻**_24_ | Transfusion medicine |
| **􀁻**_12_ | | Laboratory medicine | **􀁻**_25_ | Urology |
| **􀁻**_99_ | Other (please state):  ______________________________________________________________________ | | | |

| 14. | Would you become a physician again? | **􀁻**_00_ | No |
| --- | --- | --- | --- |
|  |  | **􀁻**_12_ | Yes |
|  |  | **􀁻**_22_ | Don't know |

| 15. | Are you currently working? (If you are on parental leave please answer „no“) | **􀁻**_0_ | No | **🢡** | **Continue with question 32** |
| --- | --- | --- | --- | --- | --- |
|  |  | **􀁻**_1_ | Yes | **🢡** | **Continue with question 16** |

With the following questions we would like to elucidate your satisfaction with your current job situation.

| 16. | Please indicate to which degree you are satisfied with your **overall** job situation. | | | | |
| --- | --- | --- | --- | --- | --- |
| **Very dissatisfied** | | **Rather dissatisfied** | **Neither nor** | **Rather satisfied** | **Very satisfied** |
| ① | | ② | ③ | ④ | ⑤ |

| 17. | Please indicate to which degree you are satisfied with the following **aspects** of your job situation: | | | | | |
| --- | --- | --- | --- | --- | --- | --- |
|  | | **Very dissatisfied** | **Rather dissatisfied** | **Neither nor** | **Rather satisfied** | **Very satisfied** |
| Work load | | ① | ② | ③ | ④ | ⑤ |
| Intellectual stimulation at work | | ① | ② | ③ | ④ | ⑤ |
| Time for family, friends, and leisure activities | | ① | ② | ③ | ④ | ⑤ |
| Stress level at work | | ① | ② | ③ | ④ | ⑤ |
| Relationship with superiors | | ① | ② | ③ | ④ | ⑤ |
| Relationship and professional exchange with colleagues | | ① | ② | ③ | ④ | ⑤ |
| Time for administrative tasks | | ① | ② | ③ | ④ | ⑤ |
| Training opportunities | | ① | ② | ③ | ④ | ⑤ |
| Career opportunities | | ① | ② | ③ | ④ | ⑤ |
| Work enjoyment | | ① | ② | ③ | ④ | ⑤ |
| Work atmosphere | | ① | ② | ③ | ④ | ⑤ |
| Social status | | ① | ② | ③ | ④ | ⑤ |
| Current income | | ① | ② | ③ | ④ | ⑤ |
| Job security | | ① | ② | ③ | ④ | ⑤ |
| Equality of women and men | | ① | ② | ③ | ④ | ⑤ |

| 18. | Are you currently mainly involved in clinical work? | | |
| --- | --- | --- | --- |
| **􀁻**_1_ | Yes, I work full-time | **🢡** | **Continue with question 19** |
| **􀁻**_2_ | Yes, I work part-time | **🢡** | **Continue with question 19** |
| **􀁻**_3_ | No, I currently work in a non-clinical setting (full-time or part-time) | **🢡** | **Continue with question 35** |

You are currently mainly involved in clinical work. We therefore ask you to please also answer the following questions regarding your satisfaction with your job situation.

| 19. | Please indicate to which degree you are satisfied with the following **aspects** of your job situation: | | | | | |
| --- | --- | --- | --- | --- | --- | --- |
|  | | **Very dissatisfied** | **Rather dissatisfied** | **Neither nor** | **Rather satisfied** | **Very satisfied** |
| Relationship with non-medical staff (nurses, doctor's assistants etc.) | | ① | ② | ③ | ④ | ⑤ |
| Relationship with patients | | ① | ② | ③ | ④ | ⑤ |
| Possibility to treat patients as you deem optimal | | ① | ② | ③ | ④ | ⑤ |
| Possibility to refer patients to specialists whenever you deem it necessary | | ① | ② | ③ | ④ | ⑤ |
| Quality of the medical care you provide | | ① | ② | ③ | ④ | ⑤ |

We would now like to know how you feel about your job situation.

| 20. | **Maslach Burnout Inventory not shown due to copyright issues** |
| --- | --- |

Please also answer the following questions regarding your job situation.

| 21. | Which position do you currently hold? | | |
| --- | --- | --- | --- |
| **Inpatient setting** | | **Outpatient setting** | |
| **􀁻**_1_ | Medical director | **􀁻**_5_ | Physician in own medical practice covering patients with statutory health insurance |
| **􀁻**_2_ | Assistant medical director | **􀁻**_6_ | Physician in own medical practice covering patients with private health insurance |
| **􀁻**_3_ | Senior physician | **􀁻**_7_ | Physician employed in medical practice |
| **􀁻**_4_ | Ward physician | **􀁻**_8_ | Physician employed in an ambulatory healthcare centre |
| **􀁻**_88_ | Other:  ________________________________ | **􀁻**_99_ | Other:  _____________________________ |
|  | |  | |
| **⮩ Please continue with question 22** | | **⮩ Please continue with question 23** | |

| 22. | You work in a hospital with | **􀁻**_1_ | More than 100 beds |
| --- | --- | --- | --- |
|  |  | **􀁻**_2_ | Less than 100 beds |

**⮩ Please continue with question 24**

| 23. | You work as a | **􀁻**_1_ | Family practitioner |
| --- | --- | --- | --- |
|  |  | **􀁻**_2_ | Specialist |

**⮩ Please continue with question 24**

| 24. | Where do you work? | **􀁻**_1_ | In an urban setting |
| --- | --- | --- | --- |
|  |  | **􀁻**_2_ | In a rural setting |

**⮩ Please continue with next question (question 25)**

In Germany there is currently a lot of debate about whether and why more and more physicians are moving abroad for clinical work. We would like to know if you have ever considered going abroad for clinical work (please note that it is only about clinical activities abroad but not about non-clinical activities such as e.g. a research stay).

| 25. | How much do you currently wish you could go abroad for clinical work? | | | | |
| --- | --- | --- | --- | --- | --- |
| **Not at all** | | **Rather not** | **Don’t know** | **Rather yes** | **Absolutely** |
| ① | | ② | ③ | ④ | ⑤ |

| 26. | Have you ever thoroughly searched possibilities for clinical work abroad? | **􀁻**_0_ | No |
| --- | --- | --- | --- |
|  |  | **􀁻**_1_ | Yes, more that 3 years ago |
|  |  | **􀁻**_2_ | Yes, during the last 3 years |

| 27. | How **likely** do you think it is that you will be going abroad for clinical work during the next 5 years …. | | | | | |
| --- | --- | --- | --- | --- | --- | --- |
|  | | **Not at all** | **Rather not** | **Don’t know** | **Rather yes** | **Definitely** |
| …for a limited period of time | | ① | ② | ③ | ④ | ⑤ |
| …for an unlimited period of time | | ① | ② | ③ | ④ | ⑤ |

| 28. | Which country, apart from Germany, would you most likely choose for working in patient care? (Please chose **only one** country)? | | |  |
| --- | --- | --- | --- | --- |
| **􀁻**_0_ | None | **􀁻**_4_ | Austria |  |
| **􀁻**_1_ | Australia | **􀁻**_5_ | Sweden |  |
| **􀁻**_2_ | France | **􀁻**_6_ | Switzerland |  |
| **􀁻**_3_ | Great Britain | **􀁻**_7_ | USA |  |
| **􀁻**_99_ | Other:  ______________________________________________________________________ | | | |

In Germany there is currently a lively discussion about whether and why more and more doctors are leaving the clinical workforce. We would like to know if you have ever considered leaving clinical work.

| 29. | How much do you currently wish you could leave clinical work? | | | | |
| --- | --- | --- | --- | --- | --- |
| **Not at all** | | **Rather not** | **Don’t know** | **Rather yes** | **Absolutely** |
| ① | | ② | ③ | ④ | ⑤ |

| 30. | Have you ever thoroughly considered which alternatives to clinical work you have? | **􀁻**_0_ | No |
| --- | --- | --- | --- |
|  |  | **􀁻**_1_ | Yes, more than 3 years ago |
|  |  | **􀁻**_2_ | Yes, during the last 3 years |

| 31. | How likely do you think it is that you will leave clinical work during the next 5 years … | | | | | |
| --- | --- | --- | --- | --- | --- | --- |
|  | | **Not at all** | **Rather not** | **Don’t know** | **Rather yes** | **Definitely** |
| …for a **limited** period of time due to **personal** reasons (e.g. parental leave, caring for relatives) | | ① | ② | ③ | ④ | ⑤ |
| … for a **limited** period of time due to **professional** reasons (e.g. research stay, teaching, professional reorientation) | | ① | ② | ③ | ④ | ⑤ |
| … for an **unlimited** period of time due to **personal** reasons (e.g. parental leave, caring for relatives) | | ① | ② | ③ | ④ | ⑤ |
| … for an **unlimited** period of time due to **professional** reasons (e.g. research stay, teaching, professional reorientation) | | ① | ② | ③ | ④ | ⑤ |

**⮩ Please continue with question 37**

You are currently not working. Please state your reasons.

| 32. | Why are you currently not working? | | |
| --- | --- | --- | --- |
| **􀁻**_1_ | I am taking care of my child(ren) |  |  |
| **􀁻**_2_ | I am taking care of other relatives |  |  |
| **􀁻**_3_ | I am seeking for a job / I am unemployed |  |  |
| **􀁻**_99_ | Other (please state):  ______________________________________________________________________ | | |

| 33. | Was your last job … | | |
| --- | --- | --- | --- |
| **􀁻**_1_ | Clinical work | **🢡** | **Please turn page and continue with question**  **35** |
| **􀁻**_2_ | Non-clinical work | **🢡** | **Please turn page and continue with question**  **35** |
| **􀁻**_0_ | I have never been working | **🢡** | **Please continue with question 34** |

| 34. | Would you prefer a clinical or non-clinical job in the future? | **􀁻**_1_ | Clinical |
| --- | --- | --- | --- |
|  |  | **􀁻**_2_ | Non-clinical |
|  |  | **􀁻**_3_ | Don’t know |
| **⮩** | If you have never been working, please finish the questionnaire here. Many thanks for your cooperation! | | |

You are currently working non-clinically or you do not work. Please answer the following questions.

| 35. | For how long have you not been in clinical work? | **􀁻**_0_ | I have never been in clinical work |
| --- | --- | --- | --- |
|  |  | _____________ year(s) | |

| 36. | Did you limit your time off from clinical work from the outset? |
| --- | --- |
| **􀁻**_0_ | No |
| **􀁻**_1_ | Yes, not longer than 12 months |
| **􀁻**_2_ | Yes, 12 months to 3 years |
| **􀁻**_3_ | Yes, more than 3 years |

**⮩ Please continue with next question (question 37)**

We will now list potential reasons to decide against clinical work.

| 37. | If you are **currently working clinically**, please think about your **present** situation.  If you are **currently working non-clinically** or if you are **currently not working**, please think about your **last time** in clinical work. Please indicate whether the respective fact applies/applied to you. Please then indicate which of the applicable reasons were/would be the **three most important ones** for you to leave clinical work. If you **have never been** in clinical work, please indicate the three most important reasons to decide against clinical work in the first place. | | | | |
| --- | --- | --- | --- | --- | --- |
|  | | **Doesn’t apply to me** | **Does apply to me** | **Three most important reasons** | |
| Poor reconciliation of work and family due to long / irregular working hours | | ⓪ | ① | 🌕 | |
| Poor reconciliation of work and family due to inadequate childcare facilities | | ⓪ | ① | 🌕 | |
| Poor reconciliation of work and private interests (hobbies, friends etc.) | | ⓪ | ① | 🌕 | |
| Cannot work clinically due to health reasons | | ⓪ | ① | 🌕 | |
| High burden due to on-call or shift services | | ⓪ | ① | 🌕 | |
| Lack of opportunities for flexible working hours (e.g. part-time jobs) | | ⓪ | ① | 🌕 | |
| Overtime hours on a regular basis | | ⓪ | ① | 🌕 | |
| High work load | | ⓪ | ① | 🌕 | |
| Bad work atmosphere | | ⓪ | ① | 🌕 | |
| Lack of cooperation among colleagues | | ⓪ | ① | 🌕 | |
| Bullying | | ⓪ | ① | 🌕 | |
| Conflicts / problems with superiors | | ⓪ | ① | 🌕 | |
| Dissatisfaction with leadership style of supervisor | | ⓪ | ① | 🌕 | |
| Lack of career opportunities | | ⓪ | ① | 🌕 | |
| Inadequate salary | | ⓪ | ① | 🌕 | |
| High degree of administrative work | | ⓪ | ① | 🌕 | |
| Lack of time for patients | | ⓪ | ① | 🌕 | |
| Formal reasons (expiration of employment contract) | | ⓪ | ① | 🌕 | |
| Professional reorientation | | ⓪ | ① | 🌕 | |
| Dissatisfaction with organization / quality of training | | ⓪ | ① | 🌕 | |
| Double burden due to research and clinical activity | | ⓪ | ① | 🌕 | |
| Discrimination of women | | ⓪ | ① | 🌕 | |
| Other:  **____________________________________________________________** | | | | | 🌕 |

| 38. | If you are currently working clinically please indicate which alternative professional field you would prefer. If you are currently working non-clinically please indicate in which professional area you are currently working.  Please indicate the area that best applies to you. | | |
| --- | --- | --- | --- |
| **􀁻**_0_ | None | **􀁻**_4_ | Medical management |
| **􀁻**_1_ | Teaching / research at colleges or universities | **􀁻**_5_ | Medical technology |
| **􀁻**_2_ | Media or publishing industry | **􀁻**_6_ | Pharmaceutical industry |
| **􀁻**_3_ | Medical computer sciences | **􀁻**_7_ | Health policy (health insurance, health authorities, etc.) |
| **􀁻**_99_ | Other:  _________________________________________________ | | |

**Thank you very much for**

**completing the questionnaire!**
